# Specific Aptamer-Based Probe for Analyzing Biomarker MCP Entry Into Singapore Grouper Iridovirus-Infected Host Cells via Clathrin-Mediated Endocytosis

**DOI:** 10.3389/fmicb.2020.01206

**Published:** 2020-06-19

**Authors:** Qing Yu, Mingzhu Liu, Siting Wu, Xinxian Wei, Hehe Xiao, Yi Yi, Hao Cheng, Shaowen Wang, Qin Zhang, Qiwei Qin, Pengfei Li

**Affiliations:** ^1^Guangxi Key Laboratory of Marine Natural Products and Combinatorial Biosynthesis Chemistry, Guangxi Beibu Gulf Marine Research Center, Guangxi Academy of Sciences, Nanning, China; ^2^Guangdong Laboratory for Lingnan Modern Agriculture, College of Marine Science, South China Agricultural University, Guangzhou, China; ^3^Guangxi Key Laboratory for Polysaccharide Materials and Modifications, Guangxi Colleges and Universities Key Laboratory of Utilization of Microbial and Botanical Resources, School of Marine Sciences and Biotechnology, Guangxi University for Nationalities, Nanning, China; ^4^Guangxi Key Laboratory of Aquatic Genetic Breeding and Healthy Aquaculture, Academy of Fishery Sciences, Nanning, China; ^5^Guangxi Key Laboratory of Green Processing of Sugar Resources, College of Biological and Chemical Engineering, Guangxi University of Science and Technology, Liuzhou, China

**Keywords:** grouper iridovirus, aptamer, endocytosis, major capsid protein, viral pathogenesis

## Abstract

Biomarkers have important roles in various physiological functions and disease pathogenesis. As a nucleocytoplasmic DNA virus, Singapore grouper iridovirus (SGIV) causes high economic losses in the mariculture industry. Aptamer-Q5-complexed major capsid protein (MCP) in the membrane of SGIV-infected cells can be used as a specific molecular probe to investigate the crucial events of MCP endocytosis into SGIV-infected host cells during viral infection. Chlorpromazine blocks clathrin-mediated endocytosis, and MCP endocytosis into SGIV-infected cells decreased significantly when the cells were pretreated with chlorpromazine. The disruption of cellular cholesterol by methyl-β-cyclodextrin also significantly reduced MCP endocytosis. In contrast, inhibitors of key regulators of caveolae/raft-dependent endocytosis and macropinocytosis, including genistein, Na^+^/H^+^ exchanger, p21-activated kinase 1 (PAK1), myosin II, Rac1 GTPase, and protein kinase C (PKC), had no effect on MCP endocytosis. The endocytosis of the biomarker MCP is dependent on low pH and cytoskeletal actin filaments, as shown with various inhibitors (chloroquine, ammonia chloride, cytochalasin D). Therefore, MCP enters SGIV-infected host cells via clathrin-mediated endocytosis, which is dependent on dynamin, cholesterol, low pH, and cytoskeletal actin filaments. This is the first report of a specific aptamer-based probe used to analyze MCP endocytosis into SGIV-infected host cells during viral infection. This method provides a convenient strategy for exploring viral pathogenesis and facilitates the development of diagnostic tools for and therapeutic approaches to viral infection.

## Introduction

Iridoviruses are large icosahedral cytoplasmic DNA viruses with circularly permuted, terminally redundant, double-stranded DNA genomes (Qin et al., 2003). The family *Iridoviridae* includes six genera: *Ranavirus*, *Iridovirus*, *Chloriridovirus*, *Lymphocystivirus*, *Megalocytivirus*, and *Decapodiridovirus* (Chinchar and Duffus, 2019). Singapore grouper iridovirus (SGIV) was first isolated from the grouper *Epinephelus tauvina*. It belongs to the genus *Ranavirus* and currently causes high economic losses in the mariculture industry (Qin et al., 2003; Xiao et al., 2019; Liu et al., 2020). Understanding the pathogenesis of SGIV is necessary to develop effective therapies against it (Yu et al., 2019a). Viral infection begins with its attachment to the host cell membrane, and it then enters the cell via specific endocytosis. In the host cell, the SGIV is transported to the replication site, where the viral genes are expressed (Seisenberger et al., 2001). Many SGIV structural genes and non-structural genes have already been studied and are related to viral replication, pathogenesis, and host cell immunity (Chinchar et al., 2009; Chinchar and Duffus, 2019). During infection, modifications appear in the host cell membranes (Verdaguer et al., 2014; Abós et al., 2015; Seeger and Mason, 2015; Yu et al., 2019a), which can potentially be used as important biomarkers of infection. Such biomarkers can be used to develop diagnostic tools and therapeutic approaches to virus infection (Yildirim et al., 2007; Ashcroft, 2019). Membrane proteins account for about 30% of the total cellular proteins and have important roles in various physiological functions (Shangguan et al., 2008). Knowledge of these biomarkers will extend our understanding of viral pathogenesis. However, little is yet known about the mechanisms underlying the entry of these biomarkers into host cells. To address this limitation, we used aptamers to investigate the crucial events of biomarker endocytosis into SGIV-infected host cells during viral infection.

Aptamers are selected by the systematic evolution of ligands with the exponential enrichment technology (SELEX) (Ellington and Szostak, 1990). Aptamers selected against different targets are synthetic oligonucleotides with different sequences and fold into distinct three-dimensional structures, binding their targets with high specificity and affinity (Yu et al., 2019b). Although they resemble antibodies in this regard, aptamers have properties that make them more useful than antibodies, such as their ease in synthesis and modification, high reproducibility, and stability. Based on these excellent qualities, aptamers are excellent molecular probes for pathogen diagnostics and therapeutics (Li et al., 2014, 2016; Wolter and Mayer, 2017; Kaur et al., 2018; Zhou et al., 2020). For example, aptamer A10 was selected against the coat protein of red-spotted grouper nervous necrosis virus (RGNNV) and was successfully used to develop an aptamer-based enzyme-linked apta-sorbent assay (ELASA) for the rapid and sensitive detection of RGNNV infection (Zhou et al., 2016, 2017). Aptamers are also used as highly specific molecular probes in pathogen pathogenesis studies (Jin et al., 2016; Yu et al., 2019a). They have been widely used to identify membrane protein biomarkers, such as alkaline phosphatase placental-like 2 (Dua et al., 2013), stress-induced phosphoprotein 1 (Van et al., 2014), and protein tyrosine kinase 7 (Shangguan et al., 2008). According to several reports, after specifically binding to biomarkers in the cell membrane, some aptamers are actively internalized into the target cell. Consequently, they can be used as delivery vehicles for the targeted delivery of drugs (Sun and Zu, 2015). For example, McNamara et al. (2006) reported the cell-type-specific delivery of small interfering RNAs (siRNA)-aptamer chimeras, which significantly increased the therapeutic efficacy of the siRNA against cancer cells. Because aptamers are easy to synthesize, they have great potential utility in targeted therapies. However, little is yet known about the mechanisms of entry of these cell biomarkers into host cells.

In a previous study, the aptamer Q5, which targets whole grouper-iridovirus-infected cells, was successfully generated with Cell-SELEX (Li et al., 2015). Aptamer Q5 has high binding affinity (24.35 nM) and recognizes SGIV-infected cells with high specificity. The target protein of aptamer Q5 on the membrane of SGIV-infected host cells was identified as the major capsid protein (MCP) of the virus (Yu et al., 2019a). MCP is the predominant structural protein of iridovirus particles and comprises about 40%–45% of the iridovirus particle polypeptides. It is used as a marker to identify and classify iridoviruses (Lin et al., 2014). MCP plays an important role in iridovirus pathogenesis, including in the formation of the iridovirus particle, the induction of the immune responses, virus–host cell interactions, antigen recognition, and the transcription of viral genes (Fu et al., 2012). After specifically binding to MCP in the cell membrane, aptamer Q5 is actively internalized into the target cells, indicating that MCP is transported by the vesicular transport system in the host cell during viral infection. Therefore, aptamer Q5 can be used as a specific probe with which to investigate the trafficking mechanism and endocytotic pathway of MCP in host cells during SGIV infection (Wickner and Schekman, 2005; Yu et al., 2019a). Briefly, aptamer Q5 first binds to the target protein (MCP) on the cell surface, and the Q5–MCP interaction activates the cellular signaling pathways, which internalize Q5–MCP through one of several endocytotic mechanisms. These include clathrin-mediated endocytosis, caveolae/raft-dependent endocytosis, macropinocytosis, and non-clathrin–caveolae/raft-dependent endocytosis (Wang et al., 2014; Huang et al., 2017). In this study, an aptamer (Q5)-based specific probe was used to investigate the trafficking mechanism and endocytotic pathway of MCP in host cells during SGIV infection.

## Materials and Methods

### Virus, Cell Lines, and Cell Culture

Grouper spleen (GS) cells were grown in Leibovitz’s L-15 medium (Gibco, Grand Island, NY, United States) containing 10% fetal bovine serum (Gibco) at 28°C. The grouper iridovirus strain (SGIV-Gx) used in this study was isolated from diseased hybrid grouper (*Epinephelus fuscoguttatus*♀ × *E. lanceolatus*♂) in Guangxi Province, China, propagated in GS cells, and stored at −80°C in the laboratory (Xiao et al., 2019).

### Virus-Infected Cells

Grouper spleen cells were cultured to 100% confluence in 12-well plates or 35-mm glass-bottom dishes (Cellvis, Hangzhou, China; catalog number D35-14-1-N) at 28°C for 24 h. After the cells were infected with SGIV-Gx at a multiplicity of infection (MOI) of 1 at 28°C for 6 h, they were then washed with phosphate-buffered saline (PBS: 10 mM Na_2_HPO_4_⋅12H_2_O, 2 mM KH_2_PO_4_, 137 mM NaCl, 1% NaN_3_) and collected for further use (Yu et al., 2019a).

### Aptamer, Reagents, and Biochemical Inhibitors

The aptamer Q5 was generated against SGIV-infected cells with Cell-SELEX in our previous study (Li et al., 2015). Aptamer Q5 was synthesized by Life Technologies, and the 5′ end of aptamer Q5 was labeled fluorescently with Cy5 (Cy5-Q5). Chlorpromazine (CPZ), methyl-β-cyclodextrin (MβCD), nystatin, genistein, dynasore, (N-ethyl-N-isopropyl)-amiloride (EIPA), ML-7, NSC23766, rottlerin, IPA-3, chloroquine (CQ), ammonia chloride (NH_4_Cl), cytochalasin D (cytoD), and nocodazole were purchased from Sigma-Aldrich (St. Louis, MO, United States). All reagents were stored and dissolved to specific concentrations according to the manufacturer’s instructions.

### Analysis of the Safe Working Concentrations of Inhibitors With Cell Viability Assays

To optimize the safe working concentration of each inhibitor, its cytotoxicity was evaluated as described previously (Liu et al., 2019). GS cells (1 × 10^4^) were cultured in a 96-well plate (Corning, New York, United States) for 24 h at 28°C, and then incubated with different concentrations of each inhibitor (15 μM CPZ, 20 μM dynasore, 2 mM MβCD, 100 μM genistein, 40 μM EIPA, 20 μM IPA-3, 20 μM ML-7, 200 μM NSC23766, 1 μM rottlerin, 20 μM CQ, 400 mM NH_4_Cl, or 6 μM cytoD) at 28°C for 4 h. Untreated GS cells were used as the control group. CCK-8 solution (20 μL; Beyotime, Shanghai, China) was added to the cells, and they were incubated at 28°C for 4 h. The absorbance at 450 nm was measured with an enzyme-linked immunosorbent assay plate reader (Thermo, Waltham, MA, United States), according to the manufacturer’s instructions. The results of three independent experiments are presented as means ± standard deviations (SD). The working concentration of each inhibitor used in this study caused no significant cytotoxicity.

### Flow-Cytometric Analysis

A flow cytometer (Beckman MoFlo XDP, Brea, CA, United States) was used to analyze the specific interaction and internalization of the Q5-MCP complex (Li et al., 2015; Yu et al., 2019a). GS cells (1 × 10^5^) were cultured to 100% confluence in 12-well plates for 24 h and then infected with SGIV-Gx (MOI = 1). At 6 h post-infection, the SGIV-infected GS cells were incubated with Cy5-Q5 (200 nM) at 4°C or 28°C. After the mixtures were washed three times with PBS, they were resuspended in 400 μL of PBS for flow-cytometric analysis, in which 20,000 events were counted. Normal GS cells incubated with aptamer Cy5-Q5 (200 nM) were used as the control. The results are presented as the means ± SD of three independent experiments.

### Confocal Fluorescence Imaging Analysis

For live-cell fluorescent imaging, 1 × 10^4^ GS cells were cultured in 35-mm glass-bottom dishes (Cellvis, Hangzhou, Zhejiang, China) and infected with SGIV-Gx (MOI = 1) for 6 h. The SGIV-infected cells were then incubated with aptamer Cy5-Q5 (200 nM) at 4°C or 28°C. After the cells were washed with serum-free phenol-red-free medium, their fluorescence was detected with laser scanning confocal microscopy (LSCM; Nikon, Tokyo, Japan). Normal GS cells incubated with aptamer Cy5-Q5 (200 nM) were used as the control.

### Effects of Inhibitors on Aptamer Cy5-Q5 Binding to MCP in Membranes of SGIV-Infected Cells

We analyzed the effects of various inhibitors on the binding of aptamer Cy5-Q5 to its target protein, MCP, in the membranes of SGIV-infected cells with flow cytometry. GS cells (1 × 10^5^) were cultured in 12-well plates at 28°C for 24 h and then infected with SGIV-Gx (MOI = 1) for 6 h. After the safe working concentration of each inhibitor was applied to the cells for 1 h, the SGIV-infected cells were incubated with aptamer Cy5-Q5 (200 nM) in the presence of each inhibitors for 1 h at 4°C. The culture supernatants were then removed, and the cells were washed twice with PBS and collected for flow-cytometric analysis (20,000 events). Normal GS cells incubated with Cy5-Q5 (200 nM) were used as the negative control; SGIV-infected cells with no inhibitor treatment were incubated with Cy5-Q5 (200 nM) and used as the positive control. The results are presented as the means ± SD of three independent experiments.

### MCP Entry Assay

The MCP entry efficiency was assessed with the protocol described by Huang et al. (2017), with some modifications. GS cells were cultured to 100% confluence in 12-well plates or 35-mm glass-bottom dishes at 28°C for 24 h and then infected with SGIV-Gx (MOI = 1) for 6 h. After pretreatment with each inhibitor at the safe working concentration for 1 h, the SGIV-infected cells were incubated with aptamer Cy5-Q5 (200 nM) in the presence of each inhibitor for 1 h at 4°C. The cultured cells were then transferred to 28°C to initiate MCP endocytosis into the SGIV-infected host cells. The internalization of the Q5-MCP complex was analyzed with flow cytometry and LSCM. Normal GS cells incubated with Cy5-Q5 (200 nM) were used as the negative control; SGIV-infected cells without any inhibitor were incubated with Cy5-Q5 (200 nM) and used as the positive control. Entry efficiency was calculated with the following equation: entry efficiency = (*X* − Control 1)/(Control 2 − Control 1) × 100%, where *X* is the flow-cytometric results for each sample of inhibitor-pretreated SGIV-infected cells incubated with Cy5-Q5 (200 nM) at 28°C; Control 1 is the flow-cytometric result for SGIV-infected cells incubated with Cy5-Q5 (200 nM) at 4°C, and Control 2 is the flow-cytometric result for SGIV-infected cells incubated with Cy5-Q5 (200 nM) at 28°C. The results of the MCP entry assay for each inhibitor are presented as the means ± SD of three independent experiments.

### Statistical Analysis

The experimental data in the study are expressed as means ± SE. Intergroup differences were analyzed with one-way analysis of variance (ANOVA), with the SPSS 13.0 statistical software (IBM, Armonk, NY, United States). A *p* value < 0.05 was considered statistically significant.

## Results

### Aptamer Cy5-Q5 Specifically Bound to SGIV-Infected Cells

**FIGURE 1:**
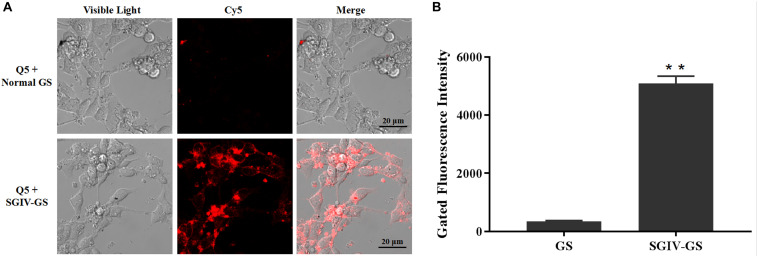
Aptamer Cy5-Q5 specifically bound to SGIV-infected cells. **(A)** LSCM showed the specific binding of Cy5-Q5 to SGIV-infected cells but not to normal GS cells. **(B)** Flow cytometry results confirmed that compared with the control group, in which aptamer Cy5-Q5 (200 nM) was incubated with normal GS cells, Cy5-Q5 specifically bound to SGIV-infected cells. *p* < 0.01 was considered statistically significant (**p < 0.01).

Confocal fluorescence imaging showed that the Cy5-labeled aptamer Q5 (Cy5-Q5) specifically bound to the surfaces of SGIV-infected cells at 4°C, but not to normal GS cells. When Cy5-Q5 was incubated with SGIV-infected cells at 4°C, the fluorescent signal was only visible around the cell plasma membrane, but not inside the cells (Figure 1A). The specific binding properties of aptamer Cy5-Q5 were confirmed with a flow-cytometric analysis (Figure 1B).

### Kinetics of Q5-MCP Complex Entry Into SGIV-Infected Cells

**FIGURE 2:**
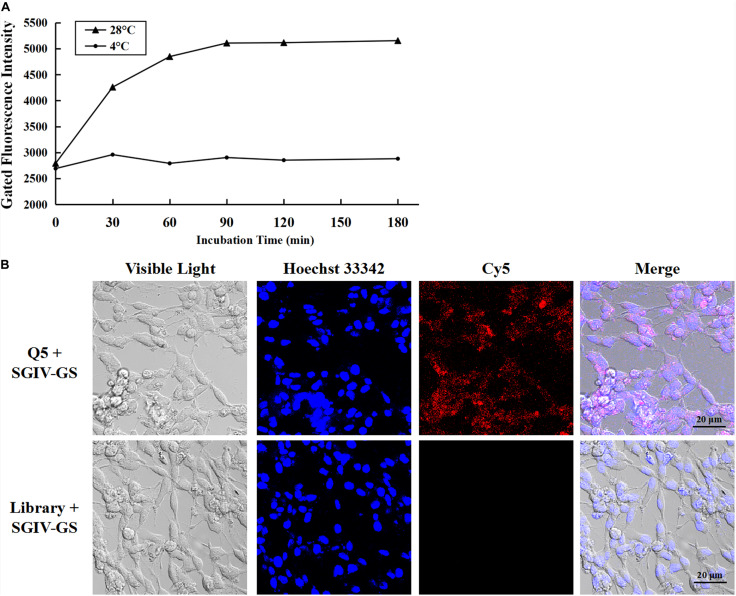
Aptamer Cy5-Q5 was used to characterize the entry process and entry kinetics of MCP into SGIV-infected cells. **(A)** Entry process and entry kinetics detected with flow cytometry. After aptamer Cy5-Q5 was incubated with SGIV-infected cells at 4°C for different periods (0, 30, 60, 90, 120, or 180 min), no significant change in fluorescence was detected with flow cytometry, indicating that aptamer Cy5-Q5 only bound to the cell plasma membrane, without endocytosis. When cells were transferred to 28°C to initiate MCP entry into SGIV-infected cells, a time-dependent increase in the fluorescent signal was detected in the cell cytoplasm with flow cytometry, which peaked at 120 min. **(B)** Cell-specific internalization of the Q5-MCP complex was detected with LSCM. After the cells were incubated at 28°C for 2 h, the fluorescent signal was observed inside the SGIV-infected cells, confirming the endocytosis of the Q5-MCP complex. The control group of SGIV-infected cells treated with Cy5-library showed no fluorescence.

As reported previously, aptamer Q5 recognizes SGIV-infected cells with high specificity and affinity, and the protein detected on the target cell membrane is the viral MCP. Therefore, aptamer Q5 can be used as a specific probe to investigate the trafficking mechanism and endocytotic pathway of MCP in host cells during SGIV infection (Yu et al., 2019a). The kinetics of the entry of the Q5-MCP complex into SGIV-infected cells were analyzed with flow cytometry (Figure 2A) and LSCM (Figure 2B). The active internalization of MCP was blocked at 4°C (Li et al., 2018). As shown in Figure 2A, after incubation with SGIV-infected cells at 4°C for different periods (0, 30, 60, 90, 120, or 180 min), no significant change in fluorescence was detected with flow cytometry, indicating that aptamer Cy5-Q5 only bound to the cell plasma membrane, but endocytosis did not occur (Figure 2A). When the cells were transferred to 28°C, the entry of the Q5-MCP complex into the SGIV-infected cells was rapidly initiated. There was a time-dependent increase in the fluorescent signal within the cell cytoplasm, which peaked at 120 min (Figure 2A). The cell-specific internalization of the Q5-MCP complex was also detected with LSCM (Figure 2B). After incubation at 28°C for 2 h, the fluorescent signal was observed inside the SGIV-infected cells, indicating the endocytosis of the Q5-MCP complex. The control group of SGIV-infected cells treated with Cy5-library showed no fluorescence (Figure 2B).

### Inhibitors Had No Effect on Cy5-Q5 Binding to SGIV-Infected Cells

**FIGURE 3:**
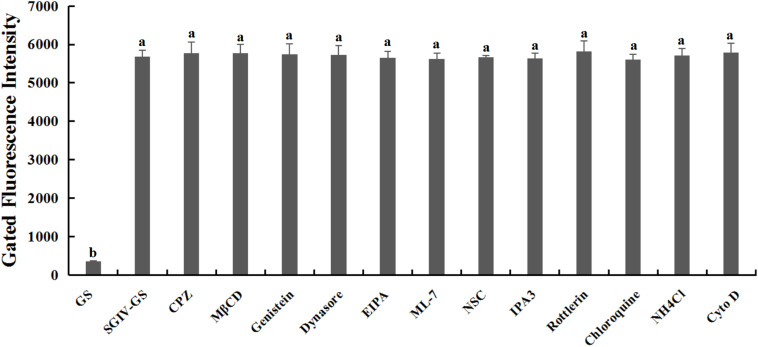
Inhibitors had no effect on Cy5-Q5 binding to SGIV-infected cells. After SGIV-infected cells were pretreated with each inhibitor at the safe working concentration for 1 h, the cells were incubated with Cy5-Q5 (200 nM) at 4°C for 1 h and then collected for flow-cytometric analysis. There was no statistically significant difference between the fluorescence signals of SGIV-infected cells pretreated with or without the inhibitors. Bars with different letters are significantly different (*p* < 0.01) based on one-way ANOVA.

Relative to the control group of normal GS cells, the GS cells incubated with the safe working concentration of each inhibitor in L-15 medium retained their normal growth, and the cell viability rate exceeded 99%, indicating that the working concentrations of the inhibitors used in this study caused no significant cytotoxicity (Supplementary Figure S1). After pretreatment with each inhibitor at its safe working concentration for 1 h, SGIV-infected cells were incubated with Cy5-Q5 (200 nM) at 4°C for 1 h and then collected for flow-cytometric analysis. The fluorescent signals did not differ significantly in the SGIV-infected cells pretreated with or without each inhibitor, indicating that the inhibitors did not interfere with the binding of Cy5-Q5 to its target on the plasma membranes of SGIV-infected cells (Figure 3).

### Clathrin-Mediated Endocytosis Mediates MCP Entry Into SGIV-Infected Cells

**FIGURE 4:**
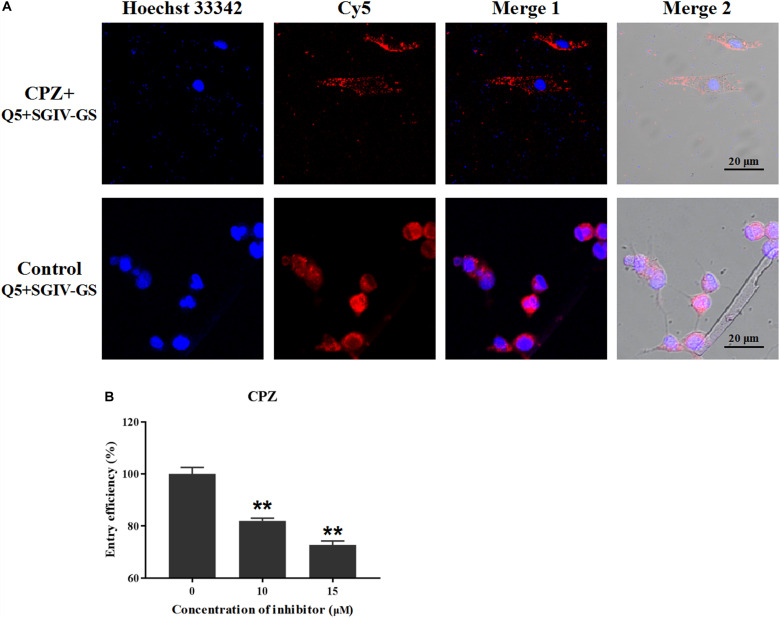
Clathrin-mediated endocytosis is involved in MCP entry into SGIV-infected cells. After SGIV-infected cells were pretreated with the inhibitor CPZ at the safe working concentration for 1 h, they were incubated with aptamer Cy5-Q5 (200 nM) at 4°C for 1 h. The culture supernatants were then removed, and the cells were washed twice with L-15 and shifted to 28°C to initiate the entry of MCP. The internalization of the Q5-MCP complex was analyzed with flow cytometry and LSCM. Normal GS cells incubated with Cy5-Q5 (200 nM) were used as the negative control; SGIV-infected cells without inhibitor but incubated with Cy5-Q5 (200 nM) were used as the positive control. **(A)** Compared with the control group, CPZ significantly reduced the fluorescent signal inside the SGIV-infected cells. **(B)** Entry efficiency of the Q5-MCP complex decreased significantly to 81.9% with 10 μM CPZ and to 72.7% with 15 μM CPZ, indicating the dose-dependent inhibitory effect of CPZ on MCP endocytosis. *p* < 0.01 was considered statistically significant (**p < 0.01).

According to our previous studies, the Q5 aptamer ultimately accumulates around the nucleus after it is efficiently internalized into SGIV-infected cells (Li et al., 2015; Yu et al., 2019a). Therefore, the effective inhibitory agents could reduce the endocytosis of Q5 and Cy5 signals in target SGIV-infected cells. Chlorpromazine (CPZ) prevents the assembly of coated pits on the cell surface, so different concentrations of CPZ were used to analyze the role of the clathrin-mediated endocytotic pathway in the entry of the Q5-MCP complex into SGIV-infected cells. As shown in Figure 4A, CPZ significantly reduced the fluorescent signal inside the SGIV-infected cells compared with that in the control group of SGIV-infected cells incubated with Cy5-Q5 without CPZ. The entry efficiency of the Q5-MCP complex also decreased significantly to 81.9% at when treated with 10 μM CPZ and to 72.7% with 15 μM CPZ, confirming the dose-dependent inhibitory effect of CPZ on Q5-MCP entry (Figure 4B).

**FIGURE 5:**
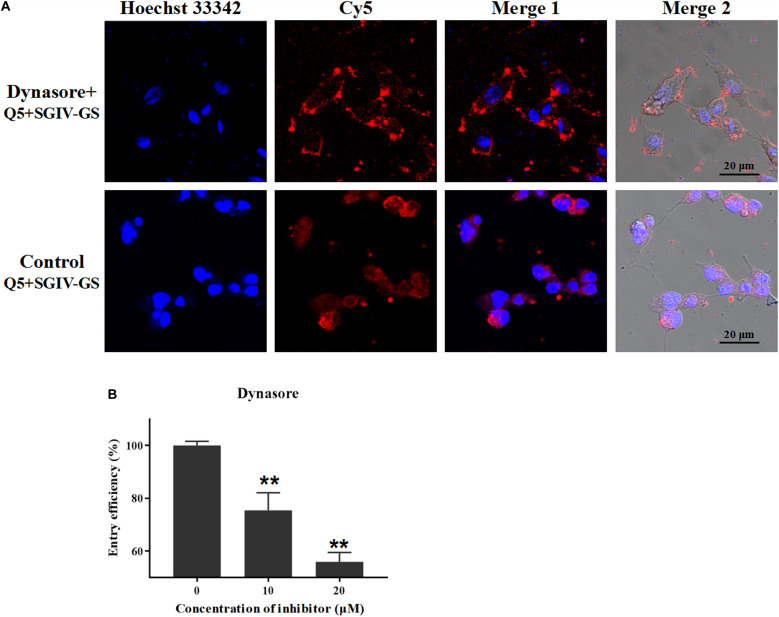
MCP endocytosis depends on dynamin. Dynamin is essential for clathrin-coated vesicle formation and membrane budding in the late stage, and dynasore is a specific inhibitor of dynamin. **(A)** The fluorescent Cy5 signal inside the SGIV-infected cells was significantly reduced in the dynasore-treated cells. **(B)** Entry efficiency of the Q5-MCP complex was greatly and dose-dependently reduced in the presence of dynasore (to 75.4% with 10 μM dynasore, to 55.9% at 20 μM dynasore). *p* < 0.01 was considered statistically significant (**p < 0.01).

Dynamin is a cellular GTPase that is essential for clathrin-coated vesicle formation and membrane budding in the late stage. Dynasore is a specific inhibitor of dynamin and rapidly blocks the formation of clathrin-coated vesicles (Abban et al., 2008). Therefore, we used different concentrations of dynasore (10 and 20 μM) to confirm the role of dynamin in the entry of the Q5-MCP complex into SGIV-infected cells. The fluorescent signal inside the SGIV-infected cells was significantly reduced in the dynasore-treated cells (Figure 5A), and the entry efficiency was greatly and dose-dependently reduced in the presence of dynasore (to 75.4% with 10 μM dynasore, to 55.9% with 20 μM dynasore) (Figure 5B). Therefore, MCP entered the SGIV-infected host cells via clathrin-mediated dynamin-dependent endocytosis.

### MCP Endocytosis Is Dependent on Membrane Cholesterol, but Not Caveolae/Raft-Dependent Endocytosis

**FIGURE 6:**
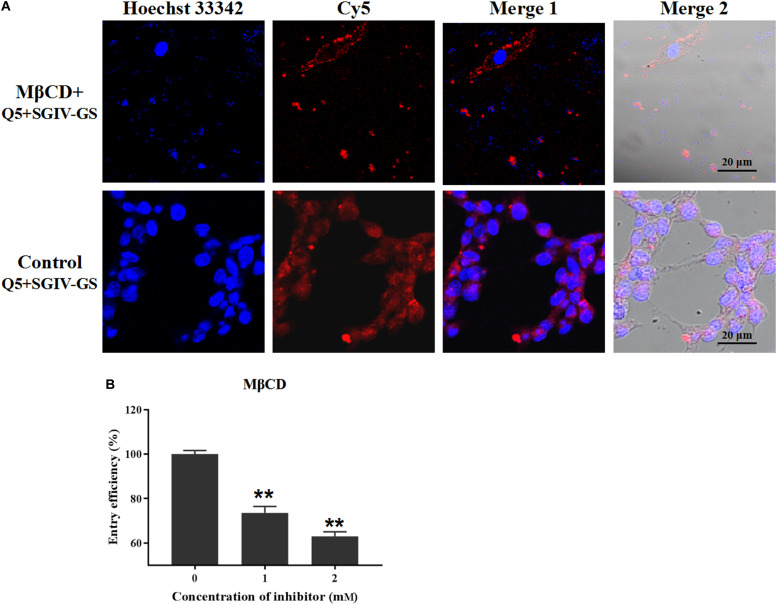
MCP endocytosis is dependent on membrane cholesterol. MβCD depletes cholesterol from the cell plasma membrane, so it was used to analyze the role of cholesterol in MCP endocytosis by SGIV-infected cells. **(A)** LSCM showed that MβCD (2 mM) significantly reduced the fluorescent Cy5 signal inside the SGIV-infected cells; most fluorescence was observed outside the MβCD-treated SGIV-infected cell membrane. **(B)** Efficiency of MCP endocytosis decreased significantly to 73.5% with 1 mM MβCD and to 63.0% with 2 mM MβCD. *p* < 0.01 was considered statistically significant (**p < 0.01).

The inhibitor MβCD depletes cholesterol from the cell plasma membrane, and cholesterol plays a critical role in caveolae/raft-dependent endocytosis (Huang et al., 2017). Therefore, we used MβCD to analyze the role of cholesterol in the entry of the Q5-MCP complex into SGIV-infected cells. As expected, MβCD (2 mM) significantly reduced the fluorescent signal inside the SGIV-infected cells, and most fluorescence was observed outside the MβCD-treated SGIV-infected cell membranes (Figure 6A). The entry efficiency of the Q5-MCP complex also decreased significantly to 73.5% after treatment with 1 mM MβCD and to 63.0% with 2 mM MβCD (Figure 6B).

**FIGURE 7:**
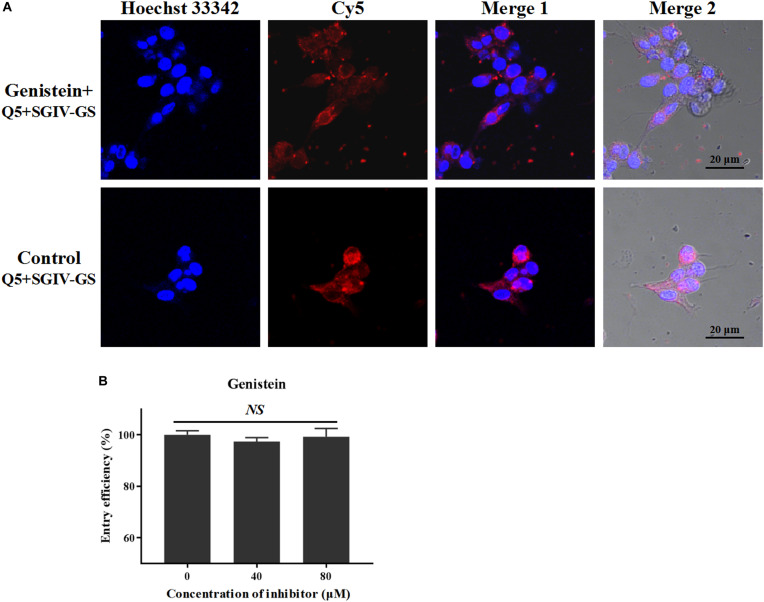
Caveolae/raft-dependent endocytosis is not involved in MCP entry during SGIV infection. Genistein is a tyrosine kinase inhibitor that blocks caveolae/raft-dependent endocytosis. **(A)** LSCM showed that even at a high concentration (100 μM), genistein did not significantly alter the fluorescent signal in SGIV-infected cells pretreated with genistein. **(B)** There was no significant difference in the entry efficiency of MCP in SGIV-infected cells pretreated with or without genistein. Genistein did not block MCP endocytosis. *NS* indicates not statistically significant.

Caveolar budding is regulated by reversible phosphorylation. Genistein is a tyrosine kinase inhibitor that blocks caveolae/raft-dependent endocytosis (Nabi, 2003). Therefore, we tested whether genistein blocks the entry of the Q5-MCP complex. As shown in Figure 7, even at a high concentration (100 μM), genistein did not block the entry of the Q5-MCP complex into SGIV-infected cells. Therefore, the entry of MCP into SGIV-infected host cells is dependent on cholesterol and membrane fluidity, but does not involve caveolae/raft-dependent endocytosis.

### MCP Endocytosis Is Independent of Macropinocytosis

**FIGURE 8:**
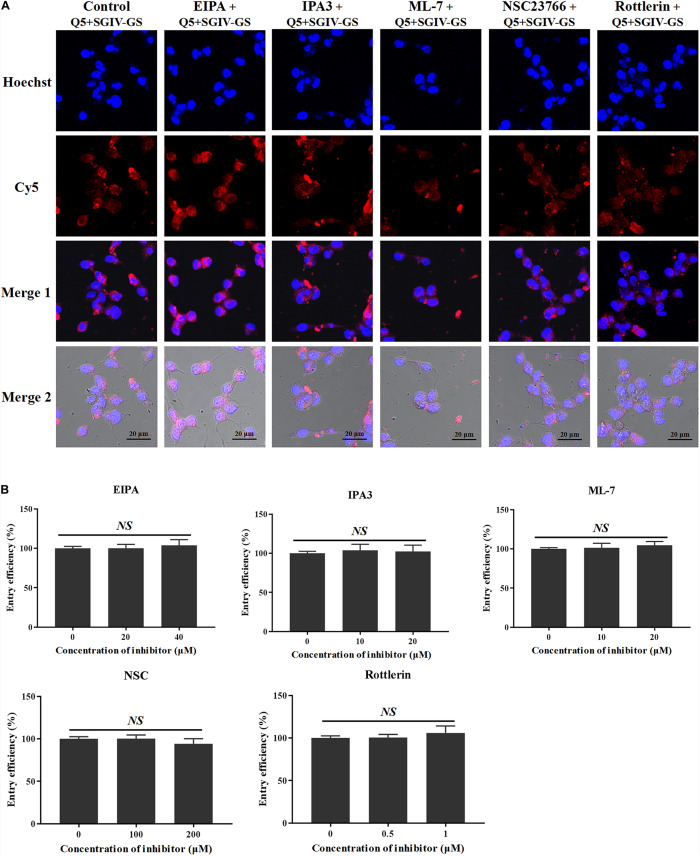
MCP endocytosis is independent of macropinocytosis. Macropinocytosis inhibitors EIPA, IPA-3, ML-7, NSC23766, and rottlerin were used to analyze the role of macropinocytosis in the endocytosis of MCP by SGIV-infected cells. **(A)** LSCM showed that even at high inhibitor concentrations (40 μM EIPA, 20 μM IPA-3, 20 μM ML-7, 200 μM NSC23766, and 1 μM rottlerin), there was no significant difference between the fluorescent signals in the SGIV-infected cells pretreated with or without the macropinocytosis inhibitors. **(B)** Entry efficiency of MCP did not differ significantly between the SGIV-infected cells pretreated with or without the macropinocytosis inhibitors. Therefore, MCP entry into SGIV-infected host cells is independent of macropinocytosis.

As well as clathrin- and caveolae/raft-dependent endocytosis, macropinocytosis has drawn increasing attention (Wang et al., 2014). Macropinocytosis inhibitors, including the Na^+^/H^+^ exchanger inhibitor EIPA, the PAK1 inhibitor IPA-3, the myosin II inhibitor ML-7, the Rac1 GTPase inhibitor NSC23766, and the protein kinase C (PKC) inhibitor rottlerin, were used to analyze the role of macropinocytosis in the entry of the Q5-MCP complex into SGIV-infected cells. As shown in Figure 8, even at high concentrations (40 μM EIPA, 20 μM IPA-3, 20 μM ML-7, 200 μM NSC23766, and 1 μM rottlerin), the macropinocytosis inhibitors did not block the entry of the Q5-MCP complex into SGIV-infected cells (Figure 8). Therefore, MCP enters SGIV-infected host cells independently of macropinocytosis.

### MCP Endocytosis Is pH Dependent

**FIGURE 9:**
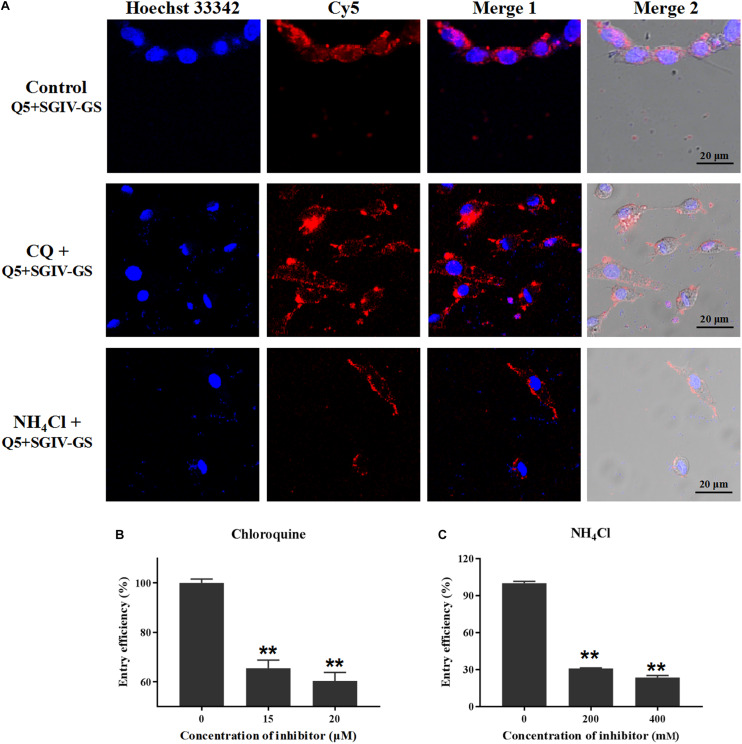
MCP endocytosis is pH dependent. Upon internalization by endocytosis, the incoming substances are usually trafficked by the pH-dependent endosomal system. CQ and NH_4_Cl inhibit endocytosis by preventing endosomal acidification, so they were used to assess whether a low endosomal pH interferes with MCP endocytosis. **(A)** LSCM showed that both CQ and NH_4_Cl reduced the internalization of the Q5-MCP complex by SGIV-infected cells. Efficiency of MCP endocytosis was significantly reduced in the presence of CQ (to 65.4% with 15 μM CQ, to 60.3% with 20 μM CQ) **(B)** or NH_4_Cl (to 30.8% with 200 mM NH_4_Cl, to 23.5% with 400 mM NH_4_Cl) **(C)** (**p < 0.01).

To assess whether low pH interferes with the entry of the Q5-MCP complex, SGIV-infected cells were pretreated with various concentrations of chloroquine (CQ) or ammonia chloride (NH_4_Cl), which act as inhibitors of endosomal acidification (Wang et al., 2014). As shown in Figure 9A, both CQ and NH_4_Cl reduced the internalization of the Q5-MCP complex into SGIV-infected cells. The entry efficiency was greatly reduced in the presence of CQ (to 65.4% with 15 μM, to 60.3% with 20 μM) (Figure 9B) or NH_4_Cl (to 30.8% with 200 mM NH_4_Cl, to 23.5% with 400 mM NH_4_Cl) (Figure 9C). Therefore, MCP endocytosis during SGIV infection is dependent upon a low endosomal pH.

### MCP Endocytosis Is Dependent on Cytoskeletal Actin Filaments

**FIGURE 10:**
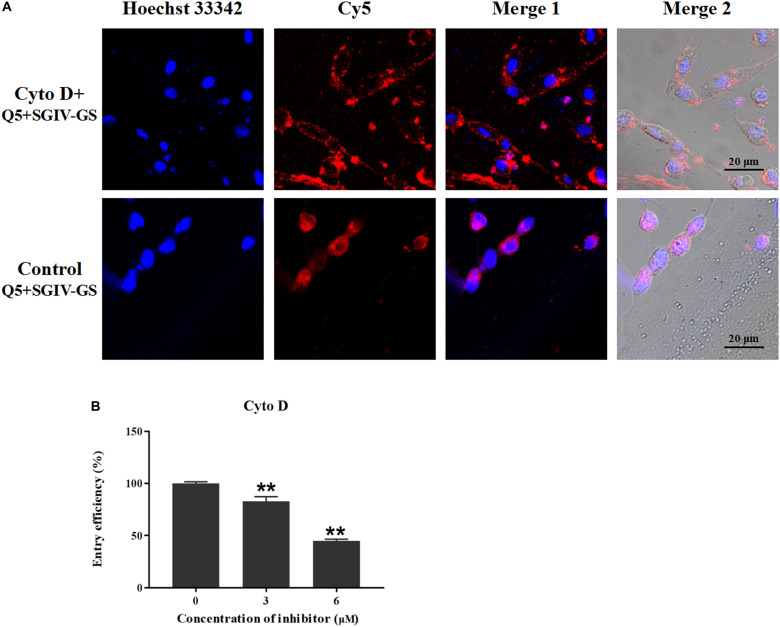
MCP endocytosis is dependent on cytoskeletal actin filaments. Cellular uptake characteristics are related to the actin cytoskeleton, which is disrupted by cytoD. Therefore, cytoD was used to analyze the role of the actin cytoskeleton in MCP endocytosis. **(A)** LSCM showed that the disruption of actin dynamics by cytoD significantly reduced the fluorescent signal inside SGIV-infected cells. **(B)** Efficiency of MCP endocytosis decreased significantly to 82.9% with 3 μM cytoD and to 44.9% with 6 μM cytoD, demonstrating that the disruption of the actin cytoskeleton significantly inhibited MCP endocytosis (**p < 0.01).

To determine the role of actin filaments in MCP endocytosis, the chemical inhibitor cytoD was used to inhibit the elongation of actin filaments (Wang et al., 2014). As shown in Figure 10, the disruption of the actin dynamics by cytoD significantly reduced the fluorescent signals inside the SGIV-infected cells. The entry efficiency also decreased significantly to 82.9% with 3 μM cytoD and 44.9% with 6 μM cytoD. Therefore, MCP endocytosis into SGIV-infected cells was inhibited by the inhibitor cytoD, indicating that MCP endocytosis during SGIV infection is dependent on cytoskeletal actin filaments.

## Discussion

During viral infection, modifications appear in the host cell membranes and are transported by the vesicular transport system of the host cell (Wickner and Schekman, 2005; Yu et al., 2019a). These modifications are associated with viral replication and host cell immunity and play critical roles in various physiological functions (Abós et al., 2015; Chinchar and Duffus, 2019). Studying the entry pathways of modifications is important in understanding viral pathogenesis and the development of diagnostic tools and therapeutic approaches to the very early critical steps of viral infection.

Because aptamers specifically recognize and bind their target molecules, they are excellent molecular probes for various biological applications, such as pathogen detection, disease diagnosis, and viral pathogenesis research (Sun and Zu, 2015; Kaur et al., 2018). Aptamer Q5 recognizes SGIV-infected cells with high specificity and affinity, and its target protein on the target cell membrane is SGIV MCP. The specific interaction between Q5 and MCP activates cellular signaling pathways, which results in the active internalization of the Q5-MCP complex via endocytotic mechanisms (Yu et al., 2019a). Therefore, aptamer Q5 can be used as a specific probe to investigate the trafficking mechanism and endocytotic pathway of MCP in host cells during SGIV infection. As demonstrated with Cy5-Q5, the entry of MCP into SGIV-infected cells was rapidly initiated at a temperature of 28°C and was followed by a time-dependent increase in the fluorescent signal inside the cells. This signal accumulated around the nucleus. The fluorescence intensity reached its maximum within 120 min, indicating that the entry of all M in the SGIV-infected cell membrane was completed within 120 min.

The endocytotic pathways predominantly include clathrin-mediated endocytosis, caveolae/raft-dependent endocytosis, macropinocytosis, non-clathrin–caveolae/raft-dependent endocytosis, and some other still poorly characterized mechanisms (Huang et al., 2017). Clathrin-mediated endocytosis is the classical endocytotic mechanism and also the best-characterized pathway. Some viruses are reported to invade their host cells via clathrin-mediated endocytosis, such as *Betanodavirus* (Huang et al., 2017), SGIV (Wang et al., 2014), and *African swine fever virus* (Hernaez and Alonso, 2010). During clathrin-mediated endocytosis, clathrin is assembled on the plasma membrane to form clathrin-coated pits (CCPs), which invaginate to form clathrin-coated vesicles containing the internalized material. CPZ is a cationic amphiphilic agent that causes the misassembly of CCPs, so it was used to determine the role of the clathrin-mediated endocytosis pathway in the entry of the Q5-MCP complex into SGIV-infected cells. In this study, the entry efficiency of the Q5-MCP complex decreased significantly and dose-dependently after CPZ treatment. Therefore, we conclude that MCP endocytosis during SGIV infection is dependent on clathrin-mediated endocytosis.

Another well-characterized endocytotic pathway is caveolae/raft-dependent endocytosis, which is an alternative to clathrin-mediated endocytosis. In this mechanism, the extracellular substance first binds to the specialized membrane domain called the “caveola,” which is composed of caveolin and associated with high levels of cholesterol. The signal cascade caused by the activation of tyrosine kinases ultimately results in the slow but efficient internalization of the substance (Doherty and McMahon, 2009). Some viruses, such as the tiger frog virus (Guo et al., 2011), infectious spleen and kidney necrosis virus (ISKNV) (Guo et al., 2012), and coronaviruses (Nomura et al., 2004), invade cells via caveola-dependent endocytosis but not clathrin-mediated endocytosis. As reported previously, cholesterol plays a critical role in caveolae/raft-dependent endocytosis (Huang et al., 2017). In this study, MβCD was used to deplete cholesterol from the plasma membrane of the host cell. As expected, MβCD significantly reduced the cholesterol during the entry of the Q5-MCP complex into SGIV-infected cells. However, the effect was insufficient to conclude that the internalization of the Q5-MCP complex is dependent upon caveolae-mediated endocytosis. According to the latest reports, cholesterol is also an essential factor in clathrin-mediated endocytosis because it regulates the fluidity of the plasma membrane, which may affect the formation of clathrin-coated vesicles (Huang et al., 2017). Some viruses, including rhesus rhadinovirus (Zhang et al., 2010), *Betanodavirus* (Huang et al., 2017), *Japanese encephalitis virus* (Das et al., 2010), and *Chikungunya virus* (Hoornweg et al., 2016), are reported to invade their host cells through cholesterol-dependent clathrin-mediated endocytosis. Therefore, whether the entry of SGIV MCP into its host cells involves caveolae/raft-dependent endocytosis warranted clarification.

Caveolae/raft-dependent endocytosis not only is sensitive to cholesterol depletion but also regulated by reversible phosphorylation (Nabi, 2003). Genistein is a natural flavonoid compound with various bioactivities and is used in the treatment of tumors (Banerjee et al., 2008), diabetes (Behloul and Wu, 2013), and skin diseases (Toutfaire et al., 2017). Genistein also acts as protein tyrosine kinase inhibitor. Previous studies of tiger frog virus (Guo et al., 2011) and ISKNV (Guo et al., 2012) have shown that genistein regulates and blocks caveolar budding during caveolae/raft-mediated endocytosis through reversible phosphorylation. Therefore, we used genistein to determine whether the entry of SGIV MCP into its host cells is dependent on caveolae/raft-dependent endocytosis. As shown in Figure 7, genistein treatment, even at a high concentration (100 μM), did not block the entry of the Q5-MCP complex into SGIV-infected cells. Therefore, we conclude that the entry of SGIV MCP into its host cells is dependent upon cholesterol but does not involve caveolae/raft-dependent endocytosis.

Macropinocytosis is an endocytotic pathway that has recently drawn increasing attention. It plays a critical role in the cellular uptake of agents (Zhou et al., 2018; Guggenheim et al., 2020), the exchange of extracellular vesicles (such as exosomes and microvesicles) between cells (Costa-Verdera et al., 2017), cellular immune defenses (Hohn et al., 2009), and viral entry (Huang et al., 2017). Both Na^+^/H^+^ exchanger activity and the functional actomyosin network are important in macropinocytosis, and many studies have reported that macropinocytosis requires signaling events and activated protein kinases such as PAK1, PKC, and GTPase Rac1. For example, the entry of SGIV into GS cells (Wang et al., 2014) and of *Echovirus 1* into polarized Caco-2 cells (Krieger et al., 2013) depends on PAK1 and PKC and is associated with macropinocytosis. Therefore, several inhibitors were used to analyze the role of macropinocytosis in MCP endocytosis during SGIV infection in this study. ML-7 was used to antagonize the phosphorylation of myosin light chain kinase, to regulate myosin II activity, which blocks the functional actomyosin network involved in macropinocytosis. NSC23766 was used to inhibit Rac1 activity, rottlerin to inhibit PKC activity, and IPA-3 to inhibit PAK1 activity in the host cells (Huang et al., 2017). However, even at high concentrations, these macropinocytosis inhibitors caused no statistically significant changes in the entry efficiency of MCP. These results suggest that macropinocytosis is not involved in MCP endocytosis.

As reported previously, cellular uptake characteristics are also associated with the cytoskeleton (Al Soraj et al., 2012; He et al., 2018). For example, cell-penetrating peptides (CPPs) have been used as vectors for the cellular delivery of therapeutic drugs. They enter cells directly across the plasma membrane and also gain access via endocytosis pathways. Al Soraj et al. (2012) found that the disruption of the actin cytoskeleton with cytoD inhibited CPP entry in the HeLa and A431 epithelial cell lines. By contrast, disruption of the actin cytoskeleton reduced the cellular uptake of dextran by HeLa cells but increased its uptake by A431 cells. This indicates that the actin cytoskeleton both promotes and interferes with endocytosis. To determine the role of the actin cytoskeleton during MCP endocytosis, cytoD was used to destroy the actin cytoskeleton by inhibiting the elongation of actin filaments. Disruption of the actin cytoskeleton significantly inhibited MCP endocytosis.

After internalization, incoming substances are usually trafficked by the pH-dependent endosomal system, which is responsible for molecular sorting, recycling, degradation, storage, processing, and transcytosis. CQ and NH_4_Cl inhibit endocytosis by preventing endosomal acidification (Guo et al., 2011). Using these inhibitors, we demonstrated that the endocytosis of the Q5-MCP complex is pH dependent.

In summary, in this study, we used a highly specific molecular probe, the aptamer Q5, to investigate the crucial events of MCP endocytosis into the host cell during SGIV infection. MCP entry into the host cell during SGIV infection is via clathrin-mediated endocytosis and is dependent on dynamin, cholesterol, low pH, and the actin cytoskeleton, but not on caveolae/raft-dependent endocytosis or macropinocytosis. This is the first time that a specific aptamer-based probe has been used to demonstrate the endocytosis of MCP during SGIV infection. The strategy developed in this study should be applicable to other types of viral infection, thus providing a convenient strategy for exploring viral pathogenesis and facilitating the development of diagnostic tools for and therapeutic approaches to viral infection. This work not only contributes greatly to our understanding of iridovirus pathogenesis but also provides an ideal molecule probe for exploring the behavior of DNA viruses in living cells.

## Data Availability Statement

All datasets generated for this study are included in the article/Supplementary Material.

## Author Contributions

PL, QQ, and QZ conceived and designed the experiments. QY, ML, SiW, and XW performed the main experiments. HX and ShW cultured the cells and performed flow cytometry analysis. YY and HC contributed to the reagents, materials, and analyzed the tools. All authors reviewed the manuscript.

## Conflict of Interest

The authors declare that the research was conducted in the absence of any commercial or financial relationships that could be construed as a potential conflict of interest.

## Supplementary Material

FIGURE S1 Identification of the safe working concentrations of the inhibitors with a cell viability assay. Relative to the control group of normal GS cells, GS cells incubated with the working concentration of each inhibitor in L15 medium retained their normal growth, and the cell viability rate exceeded 99%. These data show that the working concentrations of inhibitors used in this study caused no significant cytotoxicity. The concentrations of the inhibitors used were 15 μM CPZ, 20 μM dynasore, 2 mM MβCD, 100 μM genistein, 40 μM EIPA, 20 μM IPA-3, 20 μM ML-7, 200 μM NSC23766, 1 μM rottlerin, 20 μM CQ, 400 mM NH_4_Cl, and 6 μM cytoD. *NS* indicates not statistically significant.
