# Supplementary figures and images for: Specific Aptamer-Based Probe for Analyzing Biomarker MCP Entry Into Singapore Grouper Iridovirus-Infected Host Cells via Clathrin-Mediated Endocytosis
Source: Front Microbiol. 2020 Jun 19;11:1206. doi: 10.3389/fmicb.2020.01206 (PMC7318552; doi:10.3389/fmicb.2020.01206)

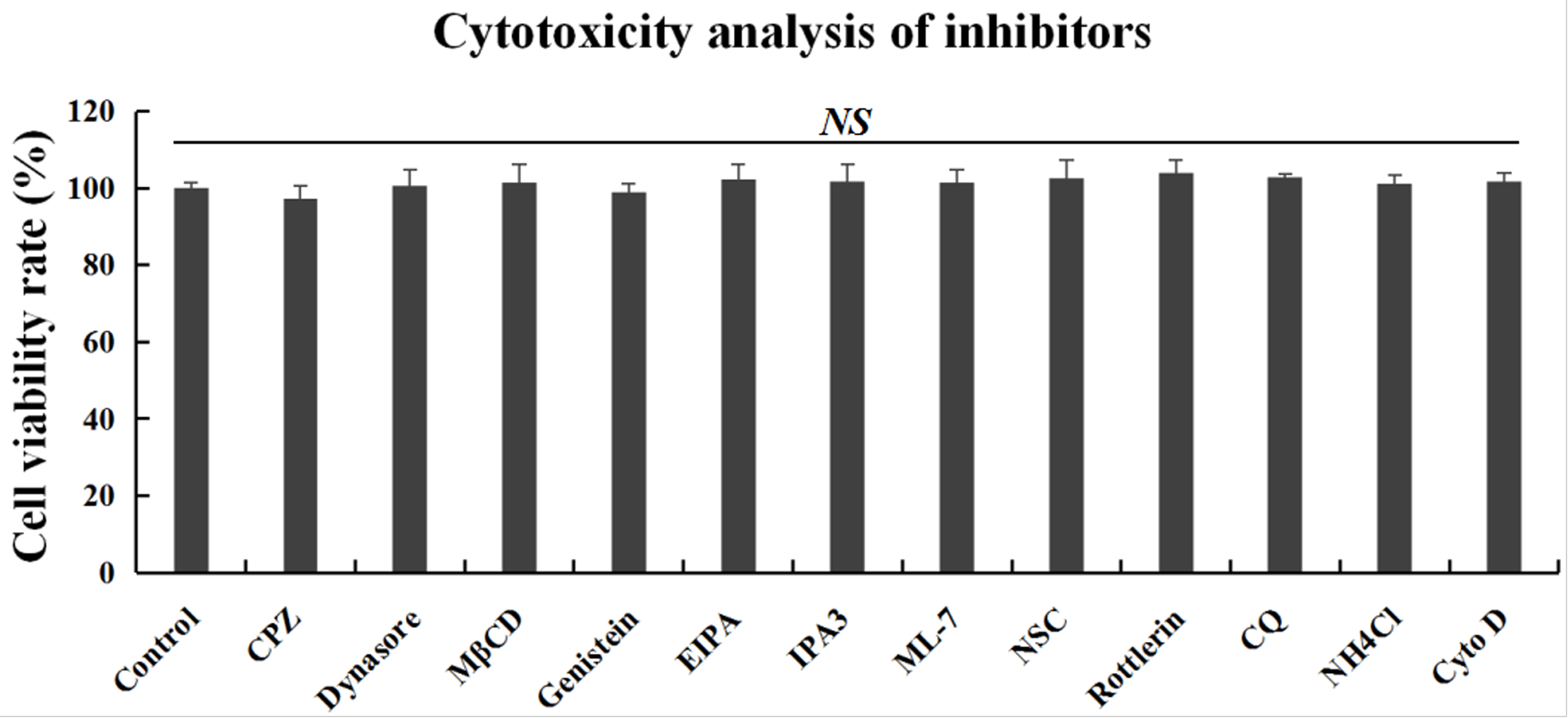

Supplement: FIGURE S1 — Identification of the safe working concentrations of the inhibitors with a cell viability assay. Relative to the control group of normal GS cells, GS cells incubated with the working concentration of each inhibitor in L15 medium retained their normal growth, and the cell viability rate exceeded 99%. These data show that the working concentrations of inhibitors used in this study caused no significant cytotoxicity. The concentrations of the inhibitors used were 15 μM CPZ, 20 μM dynasore, 2 mM MβCD, 100 μM genistein, 40 μM EIPA, 20 μM IPA-3, 20 μM ML-7, 200 μM NSC23766, 1 μM rottlerin, 20 μM CQ, 400 mM NH4Cl, and 6 μM cytoD. NS indicates not statistically significant. [file Image_1.TIF]
